# Latent profile analysis and influencing factors of psychological resilience in parents of children with autism

**DOI:** 10.3389/fpsyt.2025.1595773

**Published:** 2025-05-27

**Authors:** Chunyu Wang, Yi Shuai, Huan Wang, Zhuang Ma

**Affiliations:** ^1^ School of Nursing, Jinzhou Medical University, Jinzhou, China; ^2^ Department of Pediatric Rehabilitation, Jinzhou Rehabilitation Hospital, Jinzhou, China; ^3^ School of Physical Education and Sports Rehabilitation, Jinzhou Medical University, Jinzhou, China

**Keywords:** parents of children with autism, psychological resilience, latent profile analysis, multidimensional perceived social support, general self-efficacy, coping style

## Abstract

**Objective:**

Based on Kumpfer’s psychological resilience framework, this study aimed to explore the potential categories of psychological resilience among parents of autistic children and to analyze the influencing factors of these categories.

**Methods:**

Data were collected using a general information questionnaire, the Connor-Davidson Resilience Scale (CD-RISC), the Multidimensional Scale of Perceived Social Support (MSPSS), the General Self-Efficacy Scale (GSES), and the Simple Coping Style Questionnaire (SCSQ). From October 2023 to October 2024, a survey was conducted with 350 parents of autistic children at rehabilitation departments and hospitals in western Liaoning Province. A latent profile analysis was performed to assess the levels of psychological resilience, while univariate analysis, variance analysis, and logistic regression analysis were used to identify factors influencing the latent categories of psychological resilience.

**Results:**

The latent profile analysis identified three categories of psychological resilience among parents: low resilience – pessimistic vulnerable type (34.8%), moderate resilience – comprehensive type (42.1%), and high resilience – strong tough type (23.1%). Logistic regression analysis revealed that multidimensional perceived social support, general self-efficacy, and coping styles (both positive and negative dimensions) significantly influenced the latent categories of psychological resilience (P < 0.05).

**Conclusions:**

The psychological resilience of parents of autistic children exhibits distinct categorical characteristics. Medical staff should implement targeted and personalized interventions tailored to these categories and their influencing factors to enhance the psychological resilience of this population.

## 1 Introduction

Autism spectrum disorder (ASD) is one of the most prevalent neurodevelopmental disorders in early childhood (1). It is characterized by deficits in social communication and the presence of restricted, repetitive behaviors or interests (2). Research indicates that the global prevalence of ASD is 0.6% (3), with an incidence of 0.7% in China. The prevalence of ASD is significantly higher in boys than in girls (4). The etiology and pathogenesis of ASD remain unclear, and there is no specific treatment. Approximately 78% of children with ASD have a poor prognosis, which imposes a substantial burden on families and society, making it a major global public health concern (5). Parents of children with ASD play a crucial role in caregiving. Studies show that family caregivers of children with ASD experience higher levels of stress compared to other caregiver groups. The daily challenges of caregiving are relentless, significantly affecting the mental health (6) and quality of life of family caregivers (7). Therefore, improving the mental health of parents of children with ASD is a critical issue today.

Psychological resilience, defined as the capacity to adapt positively to adversity, was measured using the Connor-Davidson Resilience Scale (CD-RISC). With the development of positive psychology, psychological resilience has emerged as a critical research variable, garnering increasing attention. The American Psychological Association (APA) (8) conceptualizes psychological resilience from a positive psychology lens as the adaptive capacity to preserve or recover mental health when confronting major adversities like physical illness. Kumpfer’s Psychological Resilience Framework (KRF) is an integrated and widely applied model, which suggests that psychological resilience is influenced by both external environmental factors and internal resilience factors (9). Post-adversity, the interplay of internal and external factors may yield three distinct adaptation trajectories: resilient (enhanced psychological resilience), homeostatic (return to pre-stress baseline), and maladaptive (diminished psychological resilience). Studies have shown that external factors influencing psychological resilience include social support, family function (10), while internal factors include general self-efficacy (11) and coping styles (12). Recent studies further highlight the role of affective temperaments (e.g., emotional reactivity and harm avoidance) in shaping resilience patterns. For instance, Favaretto et al.synthesised three decades of evidence to demonstrate that affective temperaments modulate emotional processing and vulnerability to stress across clinical populations, underscoring the need to consider individual temperament traits in resilience research (13). Demographic and disease-related characteristics, such as gender, treatment cost, education level, and disease duration, also affect psychological resilience. Interventions targeting different levels of psychological resilience and their influencing factors can aid in the physical and mental recovery of parents of children with ASD (14). However, research on the psychological resilience of parents of children with ASD remains limited. Most studies assess psychological resilience using total scores from scales, overlooking individual differences, which complicates the implementation of targeted interventions. Latent profile analysis (LPA), a person-centered statistical method identifying homogeneous subgroups within heterogeneous populations (15), has gained prominence in organizational behavior research. This improves the accuracy and objectivity ofgrouping and helps to more intuitively and clearly demonstrate group differences, thereby providing personalized healthcare services to caregivers (16). Therefore, based on the KRF framework, this study employed LPA to identify characteristic differences in the psychological resilience of parents of children with ASD and to explore the influencing factors of these potential categories. This approach aims to expand the individual-based psychological resilience theory and provide a foundation for personalized psychological interventions.

## 2 Objects and methods

### 2.1 Survey respondents

Parents of children with autism spectrum disorder (ASD) who met the inclusion and exclusion criteria and were receiving rehabilitation training in the rehabilitation department or rehabilitation hospital of Western Liaoning District Hospital from October 2023 to October 2024 were recruited as study participants. Inclusion criteria: 1) Children with ASD: meeting the diagnostic criteria for autism as outlined in the American Diagnostic and Statistical Manual of Mental Disorders, 5th Edition (DSM-5) (17); duration of diagnosis ≥1 month; age ≤14 years. 2) Parents of children with ASD: age ≥18 years and ≤45 years; designated as the primary caregiver (one parent selected per family); providing at least 4 hours of daily care and participating in long-term caregiving; providing informed consent and willing to participate in the study. Exclusion criteria: 1) Children with ASD: presence of other significant physical or mental disorders (e.g., schizophrenia, hyperactivity, epilepsy, congenital heart disease). 2) Parents of children with ASD: experiencing severe family stress events (e.g., divorce, bereavement); diagnosed with severe mental illness or cognitive impairment; serving as a payee. Children’s ASD diagnoses were confirmed by licensed psychiatrists or clinical psychologists affiliated with the rehabilitation departments, based on DSM-5 criteria. Diagnostic information was obtained directly from the medical records provided by the participating hospitals. This study is a cross-sectional survey, with 10 items in the general data questionnaire, 3 dimensions in the perceptive social support scale, 3 dimensions in the psychological resilience scale, 10 items in the general self-efficacy scale calculated according to the number of items, and 28 variables in the simple coping style scale with 2 dimensions. According to Kendall’s sample size estimation rule (18), the sample size is at least 5-10 times the number of variables, and the loss of follow-up rate of 20% is taken into account. This study included 28 variables and determined the sample size to be 168-336 cases. A total of 368 questionnaires were sent out in this study, of which 350 were valid with complete information, and the effective recovery rate was 95.10%.

### 2.2 Survey tools

#### 2.2.1 Sociodemographic information form

A self-prepared general information questionnaire was utilized to collect demographic and contextual data. This included information on the child with ASD, such as sex, age, duration of illness, status as an only child, and monthly rehabilitation costs. Additionally, data on the parents were gathered, including age, education level, per capita monthly household income, current place of residence, and relationship to the child.

#### 2.2.2 Connor and Davidson′s resilience scale

The Connor-Davidson Resilience Scale (CD-RISC) (19) was used to assess the psychological resilience of the participants. This scale consists of 25 items divided into three dimensions: Tenacity dimension (items 11–23), Power dimension (items 1, 5, 7, 8, 9, 10, 24, 25), and Optimistic dimension (items 2, 3, 4, 6). A 4-point Likert scale was employed, with a total possible score of 100. Higher scores indicate greater psychological resilience. The Cronbach’s alpha coefficient for this scale was 0.831, demonstrating good reliability.

#### 2.2.3 The multidimensional scale of perceived social support

The scale was developed by Zimet et al. (20), consists of 12 items rated on a Likert 7-point scale, including family support (4 items), friend support (4 items), and significant others support (4 items) across three dimensions. This scale is primarily used to assess the level of perceived social support. The total score ranges from 12 to 84, with higher scores indicating better perceived social support. The Cronbach’s alpha coefficient for the scale is 0.880 (20).

#### 2.2.4 General self-efficacy scale

The General Self-Efficacy Scale was developed by Ralf Schwarzer (21) and revised into a Chinese version by Wang Caikang et al. (21). This unidimensional scale consists of 10 items, assessed using a 4-point Likert scale. The response options range from “completely incorrect” (1 point) to “completely correct” (4 points). Higher scores indicate a stronger sense of self-efficacy, with a total score range of 10 to 40. The Cronbach’s α of the original questionnaire was 0.800 (21), while the Cronbach’s α of the Chinese version of the questionnaire was 0.870 (21).

#### 2.2.5 Simplified coping style questionnaire

The Medical Coping Style Scale was developed by Folkman and Lazarus in 1984 (22) to assess the extent to which individuals use medical coping styles in response to stressful events. Xie Yaning introduced the scale to China, subsequently adapting it into the SCSQ scale through cross-cultural modifications (21). The scale consists of two dimensions: positive coping and negative coping, with a total of 20 items. The Cronbach’s alpha coefficient for the scale is 0.90 (21), indicating good reliability. Each item is rated on a 4-point Likert scale, ranging from “do not use” to “occasionally use,” “sometimes use,” and “often use,” making it a well-established tool.

### 2.3 Methods of data collection and quality control

The data of this study were collected on-site in rehabilitation hospitals and hospital rehabilitation centers in the western region of Liaoning Province, China, and were collected using electronic or paper questionnaires. The researchers rigorously screened survey participants based on the exclusion criteria and provided detailed explanations of the study’s purpose, significance, and questionnaire requirements. Questionnaires were distributed only after obtaining informed consent. Upon collection, each questionnaire was reviewed on-site for missing items, and those with random or overly consistent responses were excluded. In the recovered data of this study, the total amount of missing data was less than 5%, which was a low proportion of missing data, so the cases were deleted or processed by simple interpolation of the median or mean.

### 2.4 Statistical methods

Data analysis was conducted using Mplus 8.3 and SPSS 27.0 software. Latent profile analysis (LPA) was performed using psychological resilience (dependent variable) as a continuous variable. Common fit indices for LPA include: 1) Akaike Information Criterion (AIC), Bayesian Information Criterion (BIC), and sample-size adjusted Bayesian Information Criterion (aBIC). Lower values of AIC, BIC, and aBIC indicate better model fit. 2) Entropy, ranging from 0 to 1, evaluates the accuracy of model classification. An entropy value >0.8 signifies a classification accuracy of 90%. 3) Lo-Mendell-Rubin (LMR) test and Bootstrap Likelihood Ratio Test (BLRT): when the p-values for LMR and BLRT are <0.05, it suggests that the k-class model is superior to the (k-1)-class model. Starting from a single-class model, the number of categories was incrementally increased, and the best-fitting model was selected based on a comprehensive evaluation of all fit indices.Quantitative data conforming to a normal distribution were expressed as mean ± standard deviation (x ± s), while qualitative data were presented as frequency and percentage (%). Comparisons of basic demographics, psychological traits, and social support across latent groups were performed using the χ² test or one-way analysis of variance (ANOVA). Logistic regression analysis was applied to identify factors influencing the latent classes of psychological resilience among parents of children with ASD. Statistical significance was set at P<0.05.

## 3 Results

### 3.1 General information of survey respondents

The majority of the patients were girls, comprising 222 cases (63.4%). Most children, 209 cases (59.7%), were aged between 3 and 10 years. A total of 248 cases (70.9%) had a disease course lasting between 1 and 3 years, and 208 cases (59.4%) were the only child in their family. The monthly rehabilitation cost for most children ranged between 2,000 and 4,000 yuan, accounting for 250 cases (71.4%). Parents aged 25–35 years constituted the majority, with 249 cases (71.1%). Mothers were the primary family caregivers in 243 cases (69.4%). Regarding parental education, 157 cases (44.9%) had attained a high school or junior college level of education. Monthly family income ranged between 5,000 and 9,000 yuan in 212 cases (60.6%). Most families, 187 cases (53.4%), resided in urban or suburban areas.

### 3.2 Potential profile analysis of psychological resilience of parents of ASD children

Based on the LPA analysis of the CD-RISC across three dimensions, 1–5 potential profile models were constructed sequentially. The AIC, BIC, and aBIC values progressively decreased as the number of categories increased until the LMR index for model 5 showed no statistical significance (P > 0.05). Among all models, the AIC, BIC, and aBIC values for models 2 and 3 exhibited significant decreases, with model 3 demonstrating substantially lower values than model 2. Model 3 has good entropy (>0.9), significant LMR and BLRT tests (P <0.001), and simplicity in capturing different elastic patterns. After a comprehensive comparison, model 3 was identified as the best-fitting model, as presented in (**Table 1**).

**Table 1:** Fitting indicators of potential profile model of psychological resilience of parents of ASD children.

Model	AIC	BIC	aBIC	P		Entropy	Class probability
				LMR	BLRT		
1	22212.316	22405.213	22246.595				1.000
2	20933.479	21226.682	20985.583	<0.001	<0.001	0.934	0.720/0.280
3	20538.642	20932.152	20608.571	<0.001	<0.001	0.910	0.348/0.421/0.231
4	20390.298	20884.113	20478.052	0.007	<0.001	0.925	0.327/0.400/0.141/0.133
5	20285.514	20879.635	20391.093	0.2912	<0.001	0.928	0.154/0.197/0.378/0.138/0.133

### 3.3 Characteristics and naming of potential profile analysis of psychological resilience

**Figure 1:**
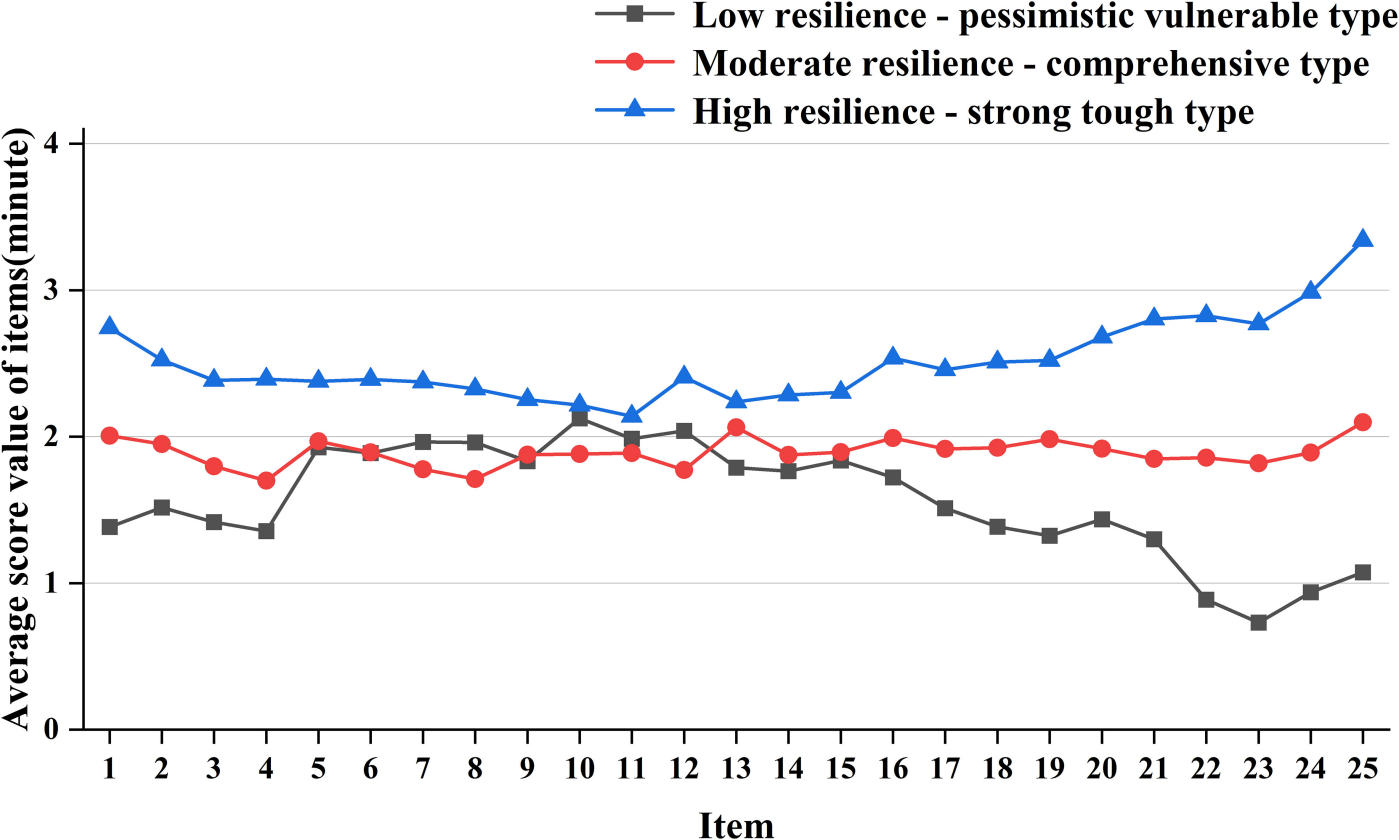
Distribution of three potential categories of psychological resilience among parents of children with ASD. (Optimistic dimension: Items 2~4, items 6; Power dimension: Item 1, item 5, item 7~10, item 24~25; Tenacity dimension: Articles 11 to 23).

This study identified three potential categories of psychological resilience among parents of children with ASD based on the LPA results. Each evaluation indicator demonstrated a good model fit, confirming individual differences in psychological resilience. Parents in Category 1 comprised 34.8% (121 cases), those in Category 2 accounted for 42.1% (150 cases), and Category 3 represented 23.1% (79 cases). A CD-RISC score line chart (**Figure 1**) was constructed to analyze the characteristics of these three categories. The attributes of each category were determined based on the fluctuations in the mean line graph of the CD-RISC dimensions.Category 1: Characterized by low scores in the optimism and tenacity dimensions, with a downward trend in these areas, it was designated as the “Low Resilience – Pessimistic Vulnerable Type.”Category 2: Exhibiting small fluctuations across dimensions, with balanced and moderate-level scores, it was labeled as the “Moderate Resilience – Comprehensive Type.”Category 3: Defined by high scores in the tenacity and power dimensions, with an overall upward trend, it was termed the “High Resilience – Strong Tough Type.”The CD-RISC scores for these categories were (39.03 ± 5.12), (47.37 ± 4.32), and (63.00 ± 6.03), respectively.

### 3.4 Single factor analysis of potential categories influencing factors of psychological resilience

The univariate analysis of general data for children with ASD and their parents revealed significant differences among the three groups (**Table 2**). Additionally, parents across the different potential categories of ASD exhibited significant differences in social support perception, self-efficacy, and coping styles (P < 0.05) (**Table 3**).

**Table 2:** Comparison of general information for children with ASD and their parents in 3 potential categories Unit: example (%).

Item	Sort	Low resilience – pessimistic vulnerable type (n = 121)	Moderate resilience – comprehensive type (n = 150)	High resilience – strong tough type (n = 79)	Statistical value	P
Gender of child	male	43 (35.5)	59 (39.3)	26 (32.9)	χ^2^=1.005	0.605
	female	78 (64.5)	91 (60.7)	53 (67.1)		
Age of child	< 3 years old	20 (16.5)	37 (24.7)	17 (21.5)	χ^2^=7.683	0.104
	3-10 years old	70 (57.9)	93 (62.0)	46 (58.2)		
	> 10 years old	31 (25.6)	20 (13.3)	16 (20.3)		
Disease course of child	< 1 year	12 (9.9)	16 (20.3)	16 (20.3)	χ^2^=18.320	<0.001
	1-3 years	78 (64.5)	111 (74.0)	59 (74.7)		
	> 3 years	31 (25.6)	23 (15.3)	4 (5.1)		
Only child	is	70 (57.9)	100 (66.7)	38 (48.1)	χ^2^=7.588	0.023
	not	51 (42.1)	50 (33.3)	41 (51.9)		
Monthly treatment cost	< 2000 RMB	1 (0.8)	14 (9.3)	21 (26.6)	χ^2^=44.695	<0.001
	2000-4000 RMB	85 (70.2)	117 (78.0)	48 (60.8)		
	> 4000 RMB	35 (28.9)	19 (12.7)	10 (12.7)		
Parental age	18-25 years old	20 (16.5)	18 (12.0)	9 (11.4)	χ^2^=2.490	0.646
	25-35 years old	80 (66.1)	111 (74.0)	58 (73.4)		
	35-45 years old	21 (17.4)	21 (14.0)	12 (15.2)		
Child patient relation	mother	89 (73.6)	119 (79.3)	35 (44.3)	χ^2^=31.398	<0.001
	father	32 (26.4)	31 (20.7)	44 (55.7)		
Parental education	Junior high school and below	37 (30.6)	25 (16.7)	11 (13.9)	χ^2^=22.075	<0.001
	High schools and colleges	52 (43.0)	78 (52.0)	27 (34.2)		
	Bachelor degree or above	32 (26.4)	47 (31.3)	41 (51.9)		
Per capita monthly household income	< 5000 RMB	41 (33.9)	27 (18.0)	10 (12.7)	χ^2^=24.075	<0.001
	5000-9000 RMB	60 (49.6)	105 (70.0)	47 (59.5)		
	> 9000 RMB	20 (16.5)	18 (12.0)	22 (27.8)		
Family place of residence	towns	57 (47.1)	82 (54.7)	48 (60.8)	χ^2^=3.742	0.154
	village	64 (52.9)	68 (45.3)	31 (39.2)		

**Table 3:** MSPSS, GSES and SCSQ scores for parents of children with 3 potential categories of ASD (x ± s).

Item		Low resilience– pessimistic vulnerable type (n = 121)	Moderate resilience – comprehensive type (n = 150)	High resilience – strong tough type (n = 79)	F	P
MSPSS		31.87 ± 7.05	40.95 ± 8.15	54.75 ± 9.04	195.181	<0.001
GSES		22.89 ± 4.81	23.85 ± 3.07	26.87 ± 3.66	26.205	<0.001
SCSQ	positive coping	20.95 ± 4.36	22.13 ± 4.35	24.53 ± 3.95	16.985	<0.001
	negative coping	10.88 ± 3.66	8.79 ± 3.63	7.81 ± 3.68	19.286	<0.001

### 3.5 Multi-factor analysis of influencing factors of potential categories of psychological resilience

The potential categories of psychological resilience among parents of children with ASD were used as the dependent variable, while indicators showing statistically significant differences in the univariate analysis were included as independent variables, with their assigned values detailed in (**Table 4**). Logistic regression analysis identified the duration of the child’s illness (independent variable), whether the child was an only child (independent variable), monthly rehabilitation costs (independent variable), parental relationship with the child (independent variable), parental education level (independent variable), family per capita monthly income (independent variable), multidimensional perceived social support (independent variable), self-efficacy (independent variable), and coping styles (independent variable) (both positive and negative coping) as significant influencing factors (P < 0.05), as shown in (**Table 5**).

**Table 4:** Variable assignment table.

Variable	Assign
Disease course of child	< 1 year=1; 1-3 years=2;> 3 years=3
Only child	Is=1;Not=2
Monthly treatment cost	< 2000 RMB=1;2000-4000 RMB=2;> 4000 RMB=3
Child patient relation	Mother=1;father=2
Parental education	Junior high school and below=1; High schools and colleges =2; Bachelor degree or above =3
Per capita monthly household income	< 5000 RMB =1; 5000-9000 RMB =2; > 9000 RMB =3
MSPSS score	Plug in the original value
GSES score	Plug in the original value
SCSQ score	Plug in the original value

**Table 5:** Multifactorial analysis of potential categories of parental psychological resilience in children with ASD.

Item				Moderate resilience – comprehensive type (n = 150)				High resilience – strong tough type (n = 79)	
		β	OR	95%CI	P	β	OR	95%CI	P
Disease course of child	< 1 year	0.477	1.611	(0.570,4.556)	0.369	1.968	7.157	(1.561,32.814)	0.011
	1-3 years	0.214	1.239	(0.549,2.794)	0.606	2.081	8.016	(2.000,32.124)	0.003
Only child	Is	0.617	1.854	(1.082,3.176)	0.025	0.142	1.152	(0.589,2.252)	0.679
Monthly treatment cost	< 2k	3.436	31.054	(3.603,267.657)	0.002	3.800	44.681	(4.745,420.783)	<0.001
	2k-4k	0.596	1.814	(0.817,4.030)	0.144	-0.369	0.691	(0.243,1.967)	0.489
Child patient relation	mother	0.428	1.534	(0.832,2.830)	0.171	-1.197	0.302	(0.149,0.611)	<0.001
Education	Junior high school and below	-0.463	0.629	(0.303,1.307)	0.214	-1.220	0.295	(0.112,0.781)	0.014
	High schools and colleges	0.108	1.114	(0.600,2.069)	0.732	-0.584	0.558	(0.262,1.187)	0.130
Per capita monthly household income	< 5k	-0.212	0.809	(0.341,1.918)	0.630	-1.274	0.280	(0.095,0.827)	0.021
	5k-9k	0.518	1.678	(0.775,3.635)	0.189	-0.574	0.563	(0.238,1.336)	0.193
MSPSS		0.177	1.193	(1.141,1.248)	<0.001	0.368	1.445	(1.343,1.556)	<0.001
GSES		0.106	1.111	(1.030,1.199)	0.006	0.392	1.480	(1.275,1.718)	<0.001
SCSQ	Positive coping	0.092	1.096	(1.024,1.174)	0.008	0.216	1.241	(1.107,1.392)	<0.001
	negative coping	-0.146	0.864	(0.809,0.924)	<0.001	-0.228	0.796	(0.731,0.867)	<0.001

The disease duration of >3 years was used as the reference. For the only child variable, “no” was taken as the reference category. Monthly rehabilitation costs >4000 RMB were used as the reference. The child’s relationship with the father served as the reference category. Parental education level was referenced to a bachelor’s degree or higher. A per capita monthly household income >9000 RMB was the reference, with “k” representing one thousand RMB.

## 4 Discussions

### 4.1 The psychological resilience of parents of ASD children can be divided into three potential categories, and the proportion of parents in the moderate resilience – comprehensive type is the highest

Based on the LPA results, three potential categories were identified, each demonstrating a good model fit and highlighting individual differences in the psychological resilience of parents of children with ASD. The psychological resilience score of the low resilience – pessimistic vulnerable type (34.8%) was 39.03 ± 5.12. This subgroup exhibited maladaptive reorganization per Kumpfer’s Resilience Framework (KRF), where biopsychosocial stressors impeded coping capacity development, culminating in compromised psychological resilience. The moderate resilience – comprehensive type (42.1%) had a resilience score of 47.37 ± 4.32, indicating a moderate level of resilience. This type, the largest proportion of participants, represents a key target for intervention and aligns with the “dynamic balance reorganization” in the KRF framework. These parents exhibit some capacity for positive adjustment but remain vulnerable to external stressors. Targeted guidance should be provided to alleviate their concerns about the disease and its treatment, support symptom management, and promote psychological resilience. Additionally, it is crucial to monitor their psychological dynamics to prevent regression into the low- resilience group. The high resilience – strong tough type (23.1%) scored above the moderate level of resilience. These parents exhibit strong willpower, reject reliance on intuition or fate, and actively grow from adversity, aligning with the “resilience restructuring” in the KRF framework. Healthcare providers can facilitate peer support activities, encouraging these parents to share their experiences to boost the parenting confidence of others while also maintaining their own high level of resilience.

### 4.2 Analysis of influencing factors of potential categories of parents of children with ASD

#### 4.2.1 Parents with moderate resilience – comprehensive type and high resilience – strong tough type have higher awareness of social support

The findings of this study suggest that high levels of social support may predict membership in the high resilience – strong tough type, with statistically significant differences observed compared to parents in the low resilience – pessimistic vulnerable type and the moderate resilience – comprehensive type (P<0.05). Previous research has identified social support as a critical predictor of loneliness, with higher levels of social support negatively correlated with loneliness (23). Social support has been shown to mitigate loneliness to some extent, thereby enhancing mental health. As an accessible and effective external resource, social support plays a vital role in psychological adjustment during stress (24), contributing to improved psychological resilience among parents of children with ASD. Additionally, it has been demonstrated to alleviate caregiver burden and reduce perceived stress among family caregivers (25). Healthcare professionals should prioritize the development of robust social support systems for families of children with ASD, establishing a foundation for their physical and mental well-being.

#### 4.2.2 Parents with low resilience – pessimistic vulnerable type have lower self-efficacy

Self-efficacy was used as a predictor in logistic regression analysis. The results of this study revealed a statistically significant difference in the GESE scores among parents of children with ASD across the three groups (P<0.05). Regression analysis indicated that, compared to the other two categories, parents with low self-efficacy were more likely to belong to the low resilience – pessimistic vulnerable type. Previous studies have demonstrated that self-efficacy plays a significant role in regulating the relationship between stress (26) and mental health (11). Self-efficacy refers to an individual’s confidence in their ability to utilize their skills to achieve success in a given situation (27). The research shows that the improvement of self-efficacy can improve the level of psychological resilience, which is consistent with the results of this study (28). When parents of children with ASD possess high self-efficacy, they are confident in their ability to solve problems, exhibit greater resilience, and demonstrate a stronger capacity to persevere in the face of adversity. Other studies have found that when parents feel more involved in their children’s treatments and more satisfied with the training they receive, they experience an increase in parenting self-efficacy (29). Furthermore, we observed that stress related to parenting a child with ASD diminishes ASD-specific parenting self-efficacy. Therefore, healthcare professionals can support parents by fostering greater confidence in their ability to manage the challenges of raising a child with ASD. This can be achieved by increasing their involvement in treatment and providing more satisfying intervention-related training. Additionally, addressing the challenges and stress that parents face, and helping them identify coping strategies, can further enhance their self-efficacy.

#### 4.2.3 Parents with high resilience – strong tough type adopt more positive coping styles and less negative coping styles

Coping styles were used as predictors in logistic regression analysis. Parents of children with ASD across the three categories demonstrated statistically significant differences in the positive and negative coping dimensions of the SCSQ (P<0.05). Regression analysis revealed that, compared to the other two categories, parents with high resilience – strong tough type were more likely to adopt positive coping strategies. Research has shown a link between coping strategies, stress, and mental health (30), and positive coping strategies have been found to promote mental health (31). This is consistent with the results of this study. When faced with challenges, parents who adopt positive coping methods actively seek support from family, healthcare, and the broader community, enabling them to effectively manage parenting stress. In contrast, parents who rely on yielding coping methods tend to experience greater uncertainty about the child’s condition and often feel powerless in the face of the prognosis and treatment outcomes. This mindset, characterized by resignation and fatalism, is associated with increased negative emotions and a higher likelihood of negative outcomes. It is recommended that healthcare professionals consider parents’ coping styles and develop targeted interventions based on their adaptive responses to reduce reliance on negative coping. Parents of children with ASD should be encouraged to minimize avoidance coping and focus on adopting more positive coping strategies.

#### 4.2.4 The course of the child’s disease, whether the child is the only child, the monthly cost of rehabilitation treatment, the relationship with the child, the educational level of the parents and the per capita monthly income of the family affect the potential types of psychological resilience of the parents of ASD children

This study found that shorter disease duration was associated with higher psychological resilience in parents, specifically the “ high resilience – strong tough type”. The duration of treatment emerged as a positive factor influencing the caregiving burden for parents of children with ASD. This may be due to the increasing psychological burden as children with ASD age and undergo longer treatments. Previous research has identified disease duration as a key factor influencing patients’ anxiety and depression scores (32). Additionally, whether the child was an only child, the monthly rehabilitation costs, and per capita household income were significant predictors of the potential categories of parental psychological resilience. The prolonged rehabilitation period and high costs of treating ASD place a greater economic strain on families, leading to an increased caregiving burden. Other studies have shown that families with autistic children have higher financial stress than families with normal children, and therefore have higher levels of caregiving burden (33). Conversely, families with higher incomes possess greater economic resources and more options for managing these challenges. This study also indicated that the caregiver’s gender significantly influenced psychological resilience levels, with mothers more likely to belong to the “ low resilience – pessimistic vulnerable type” and “ moderate resilience – comprehensive type”. This is consistent with research by scholars such as Herrero R that women perceive more burden and show poorer mental health than men (34). The overrepresentation of fathers in highly resilient groups may reflect the cultural expectations of Chinese families, where fathers often assume the role of financial provider, potentially cushioning caregiving stress through socioeconomic stability. In addition, men tend to adopt problem-focused coping strategies, which are consistent with the “tough” image. Furthermore, there were differences in parental educational levels among the ASD groups (P < 0.05). Parents in the low resilience – pessimistic vulnerable type tended to have a junior high school education or lower, while those in the other two categories had at least a high school education. Parents with higher educational levels are better able to access information through various channels. Thus, it is essential to employ diverse communication strategies to engage parents with lower educational levels, enhance their understanding of the disease and rehabilitation plans, and alleviate confusion and helplessness, ultimately improving their psychological resilience.

#### 4.2.5 Identify the clinical and psychological implications of 3 potential categories of parental resilience in children with ASD

Clinically, our findings advocate resilience-based screening to identify at-risk parents and to provide stratified interventions: for example, strengthening social support for parents with low and moderate resilience types, thereby promoting an increase in their self-efficacy to positively face the disease; It is also necessary to strengthen the care of high resilience – strong tough type, so that they avoid turning to low and moderate resilience types.

#### 4.2.6 Limitations

However, this study is limited by its focus solely on parents of children with ASD in Western Liaoning, which affects the representativeness of the sample. The KRF framework involves numerous complex factors (35), and the study does not examine all contributing factors or the causal relationships between them. Additionally, a cross-sectional design does not allow for observation of the psychological resilience trajectory over the course of the disease. Future research should employ longitudinal studies to explore the dynamic interplay of psychological resilience and its predictive factors among parents of children with ASD. Moreover, high-quality randomized controlled trials are needed to assess personalized intervention strategies, providing evidence to improve care for children with ASD and their parents. Our findings do not take into account personality or temperament variables that influence emotional resilience and may help explain inter-individual differences unexplained by sociodemographic or coping factors. Future studies need to consult the data to consider the influencing factors of psychological resilience more comprehensively.

## 5 Conclusions

This study identified three potential categories of parents of children with ASD through potential profile analysis, with the highest proportion found in the moderate resilience – comprehensive type. Additionally, based on the KRF framework, the internal and external factors influencing the different categories were examined, revealing that self-efficacy, understanding of social support, coping styles, and other factors affect psychological resilience levels. Future research should prioritize resilience-based screening tools and randomized trials to evaluate personalized interventions, such as disposition-information coping training. Such efforts can shift caregiver support from passive to proactive, ultimately improving outcomes for children with autism and their families.

## Data Availability

The original contributions presented in the study are included in the article/supplementary material. Further inquiries can be directed to the corresponding authors.
